# Microstructural White Matter Changes Underlying Cognitive and Behavioural Impairment in ALS – An *In Vivo* Study Using DTI

**DOI:** 10.1371/journal.pone.0114543

**Published:** 2014-12-11

**Authors:** Elisabeth Kasper, Christina Schuster, Judith Machts, Joern Kaufmann, Daniel Bittner, Stefan Vielhaber, Reiner Benecke, Stefan Teipel, Johannes Prudlo

**Affiliations:** 1 Department of Psychosomatic Medicine, University of Rostock, Rostock, Germany; 2 DZNE German Centre for Neurodegenerative Diseases, Site Rostock, Rostock, Germany; 3 DZNE German Centre for Neurodegenerative Diseases, Site Magdeburg, Magdeburg, Germany; 4 Department of Neurology, Otto-von-Guericke-University, Magdeburg, Germany; 5 Department of Neurology, University of Rostock, Rostock, Germany; University of Minnesota, United States of America

## Abstract

**Background:**

A relevant fraction of patients with amyotrophic lateral sclerosis (ALS) exhibit a fronto-temporal pattern of cognitive and behavioural disturbances with pronounced deficits in executive functioning and cognitive control of behaviour. Structural imaging shows a decline in fronto-temporal brain areas, but most brain imaging studies did not evaluate cognitive status. We investigated microstructural white matter changes underlying cognitive impairment using diffusion tensor imaging (DTI) in a large cohort of ALS patients.

**Methods:**

We assessed 72 non-demented ALS patients and 65 matched healthy control subjects using a comprehensive neuropsychological test battery and DTI. We compared DTI measures of fiber tract integrity using tract-based spatial statistics among ALS patients with and without cognitive impairment and healthy controls. Neuropsychological performance and behavioural measures were correlated with DTI measures.

**Results:**

Patients without cognitive impairment demonstrated white matter changes predominantly in motor tracts, including the corticospinal tract and the body of corpus callosum. Those with impairments (ca. 30%) additionally presented significant white matter alterations in extra-motor regions, particularly the frontal lobe. Executive and memory performance and behavioural measures were correlated with fiber tract integrity in large association tracts.

**Conclusion:**

In non-demented cognitively impaired ALS patients, white matter changes measured by DTI are related to disturbances of executive and memory functions, including prefrontal and temporal regions. In a group comparison, DTI is able to observe differences between cognitively unimpaired and impaired ALS patients.

## Introduction

Amyotrophic lateral sclerosis (ALS) is a neurodegenerative disease primarily characterised by a degeneration of upper and lower motor neurons [1]. Symptoms are not restricted to the motor system, but cognitive impairments and behavioural abnormalities form a relevant part of the clinical picture [2], [3]. The cognitive impairment follows a frontal-temporal pattern, with predominant deficits in executive functions and language [2], [4]. Memory impairments have also been reported but at a lower prevalence [5]. Behavioural changes in ALS are characterised by symptoms of apathy [6], and, less frequently, disinhibition and disorganisation [3]. In line with these cognitive-behavioural changes, structural brain imaging studies have demonstrated grey matter atrophy [7], [8] and white matter damage [9], [10] in fronto-temporal areas that extend beyond the motor system.

Until now, only a few imaging studies using DTI or volumetric MRI have explicitly studied the relationship between cognitive-behavioural symptoms and structural brain changes in ALS. These studies used one of two analytic approaches: group comparisons or regression analyses with cognitive tests as predictor variables. The group comparisons contrasted ALS patients with and without cognitive impairment and healthy controls (15–18), indicating both grey matter atrophy and white matter changes in ALS compared with healthy controls; nevertheless, direct comparison between cognitively-impaired and cognitively non-impaired ALS-patients had yielded no effect in previous studies [11]–[15]. This absence of an effect is difficult to interpret because few subjects (n<30) were included in these comparisons.

Correlation analyses using DTI have shown a positive association between white matter changes in frontal and temporal regions and cognitive impairment [11], [12], [15], [16]. Likewise, Pettit and colleagues [17] investigated white matter alterations underlying executive dysfunctioning in ALS using DTI, finding that reduced performance in a speed-independent dual-task paradigm was correlated with prefrontal tract integrity (i.e. anterior cingulate, anterior thalamic radiation, and uncinated fasciculus). Furthermore, Barbagello et al. [16] found a correlation between performance on neuropsychological tasks of executive functions and reduced mean diffusivity (MD) within frontal–subcortical circuits in ALS patients measuring by DTI. In addition, two functional magnetic resonance imaging (fMRI) studies have demonstrated a negative correlation between performance in executive tasks and cortical activation in frontal areas [18], [19]. However, the findings are not restricted to executive functions. Relations between performance on verbal memory tasks and widespread white matter changes were also described by Christidi et al. 2013 [20].

To our knowledge, only three imaging studies have investigated the structural basis of behavioural symptoms in ALS, two identifying correlations between apathy and frontal grey and white matter changes [21], [22]. Trojsi and colleagues [23] found correlations between behavioural scores and white matter integrity, but without a specification of individual behavioural domains.

Previous studies have elucidated important aspects of the complex relationship between brain structural integrity, cognition, and behaviour in ALS. All studies, however, involved relatively small sample sizes (<30) limiting the generalizability of those findings.

In this study, we address this limitation by examining a relatively large group of cognitively and behaviourally well-characterised ALS patients (N = 72), none of whom had dementia. We determined the association of cognitive impairment and behavioural changes with the integrity of subcortical fiber tracts using DTI with tract-based spatial statistics, providing unbiased access to the major cerebral fiber tracts. We sought to establish whether ALS patients with cognitive deficits showed distinct white matter characteristics compared with patients without cognitive deficits and, if so, to characterise these in relation to cognitive impairment. We employed a wide range of diffusion indices to provide a comprehensive characterisation of white matter microstructural integrity.

## Methods

### Participants

We analysed 72 patients recruited between 04/2011 and 12/2012 from the university hospitals in Rostock and Magdeburg, Germany. All patients were diagnosed according to the revised El Escorial criteria [24] and characterised using the ALS Functional Rating Scale-Revised (ALSFRS-R) [25]. Premorbid verbal intelligence was measured by a vocabulary test (Wortschatztest WST [26]). A healthy control group (HC) of 65 participants matched by age, gender, education, and intelligence was recruited through public advertisements.

Looking at the distribution of participants between both sites, healthy controls differed significantly in term of age (p = 0.036). Patients were matched on most clinical and demographical parameters. They only differed significantly in terms of IQ between both sites (p = 0.046) as well as in the distribution of the phenotypes (p = 0.047) and hence in the distribution of El Escorial Criteria (p = 0.023).

All of the participants received a clinical and neurological examination. All participants donated a blood sample to determine routine laboratory values. Exclusion criteria for all subjects were a history of brain injury, epilepsy, or psychiatric illness. In addition, subjects were required to have no diagnosis of fronto-temporal dementia, according to criteria by Raskovsky et al. [27] and Gorno-Tempini et al. [28]. All MRI scans were visually inspected by a radiologist to rule out major neuropathologies, such as tumours, stroke, or advanced white matter disease. In the control group, cognitive decline was assessed by means of the Montreal Cognitive Assessment (MoCA) [29], with a cut-off value of 26 (max. 30). The study was conducted according to the Declaration of Helsinki and had been approved by the local medical ethics committee at each site, with all participants providing written informed consent.

The demographical data and clinical characteristics are reported in Table 1.

**Table 1:** Sociodemographic and clinical data of all participants.

	ALS (N = 72)		HC (N = 65)		
	Mean (SD)	Range	Mean (SD)	Range	p
Age (years)	**59.3** (9.93)	32–82	**60.1** (10.44)	33–82	0.611
Gender (N male/female)	**46/26**		**38/27**		0.515
Handedness (N right/left/amb)	**69/3/0**		**61/2/2**		0.320
Education (years)	**13.1** (2.45)	9–21	**13.4** (1.86)	10–17	0.116
IQ (WST)	**102.7** (11.39)	79–139	**104.2** (6.31)	92–118	0.195
ALSFRS-R	**36.5** (6.39)	14–46			
Disease duration (months)	**30.5** (36.67)	3–272			
El Escorial (N NA/poss/prob/def)	**11/17/23/21**				
Phenotype ALS (N class/UMN/LMN)	**48/12/12**				

HC  =  Healthy Controls; NA  =  not assigned; UMN  =  Upper Motor Neuron variant; LMN  =  Lower Motor Neuron variant; SD  =  standard deviation; p  =  Level of significance from U-tests for independent samples and univariate analyses of variance (ANOVAs).

### Data acquisition

#### Neuropsychological assessment

All subjects underwent a neuropsychological test battery assessing executive functioning/attention, memory, and language and visuo-spatial abilities, administered by two psychologists from each site. The description of tasks and dependent variables is presented in Table 2. To account for patients' speech and motor impairments, speed-independent (e.g. ratios) or speed-adjusted parameters (e.g. fluency index [30]) were implemented and computerised test variants or choice-reaction tasks were employed. The Frontal System Behaviour Scale (FrSBe) was used to assess behavioural abnormalities.

**Table 2:** Description of tasks and dependent variables of the neuropsychological test battery.

Test	Dependent variables
**Trail – Making Test **[50]	TMT: Ratio of condition B and condition A
**STROOP-Paradigm **[51]	Stroop-I: Ratio of medians of Condition B^1^ and Condition A^1^
	Stroop-C: Ratio of medians of Condition C^1^ and Condition A
	Stroop-E: Total errors of Condition B and C
**Regensburg Word Fluency Test** 2 [52]	Number of words starting with letter “K” (RWT-K)
	Number of words starting with letter “G” and “R” in alteration (RWT-G/R)
	Number of animals (RWT-A)
	Number of sports and fruits in alteration (RWT-S/F)
**Tower of London** 3 [53]	ToL-E: Number of rule breakings
	ToL-M: Ratio of number of moves and number of correct trials
**Verbal Learning and Memory Test (VLMT) **[54]**/California Verbal Learning Test-Short Form (CVLT) **[55]	VM1: Trial 1
	VM2: Trial 5 (VLMT)/trial 4 (CVLT)
	VM3: Total of Trial 1-5 (VLMT)/total of trial 1-4 (CVLT)
	VMIR: Immediate recall - percentage of remembered items after interference trial (both tests merged)
	VMDR: Delayed recall - percentage of remembered items after long delay (both tests merged)
	VMR: Recognition - percentage of correctly recognized words of all affirmative words (both tests merged)
**Wechsler Memory Scale – Revised **[56]	DSF: Digit span forward^2^
	DSB: Digit span backward^2^
	VPA-I: Visual paired associates I^3^
	VPA-II-%: Visual paired associates II 3: recognition paradigm - percentage of correct recognized items
**Bogenhausen Semantic Test (BOSU) **[57]	BOSU-P: Sorting objects according to semantic features
	BOSU-N: Naming of all items
**Rey Complex Figure Test (RCFT) **[58]	RCFT: Copy trial
**Frontal System Behaviour Scale (FrSBe)** 4 [59]	FrSBe-A: Apathy
	FrSBe-D: Disinhibition
	FrSBe-E: Executive Dysfunction

1 Condition A: response to grey printed colour words; Condition B (inhibition): response to colour words with conflict of content and ink; Condition C (congruence): decision on conflict or not of word content and ink.

2 spoken/written; 1 minute generation time; use of indices.

3 computerised version.

4 Current ratings by the patients themselves (S) and by a family member (F).

#### MRI acquisition

Data were acquired in Rostock or Magdeburg with 3T Siemens MAGNETOM Verio scanners (software package: Syngo version MR B17) using 32-channel phased array head coils (Siemens, Erlangen, Germany). Identical settings and parameters were used at both sites. The orientation of the diffusion-weighted scans was required to be parallel to the anterior to posterior commissure plane. Both sites successfully conducted phantom scans to test system performance and ensure quality. Within the ACR phantom test, both sites fulfilled common criteria [31] regarding all test parameters (geometric accuracy, high contrast spatial resolution, slice thickness accuracy, slice position accuracy, image intensity uniformity, percent signal ghosting and low contrast object detectability). Within the DWI phantom scan test [32] the approximate error was calculated, revealing an error rate from 2 to 6 percent for fractional anisotropy values.

The diffusion-weighted images were acquired using a twice refocused, spin-echo echoplanar: TE = 81 ms, TR = 12700 ms, parallel imaging with GRAPPA factor = 3, voxel size = 2.0×2.0×2.0 mm^3^, acquisition matrix = 128×128, 72 slices, 1 non-diffusion weighted and 30 non-collinear diffusion gradient directions from Siemens MDDW mode, b-value = 1000 s/mm^2^, two averages, total scan time = 14 min: 10 s.

### Data analysis

#### Neuropsychological data

For the cognitive categorisation we used the classification criteria for ALS-patients by Strong et al. and Phukan et al. [33]–[35]. Their approach defines abnormal performance on a task as a score that is two or more standard deviations below the mean for healthy controls. Accordingly, the domain of executive functions was considered to be impaired when patients scored abnormal on at least 2 distinct executive tasks. The memory domain was considered to be impaired when patients scored abnormal on at least 3 of 9 memory variables. The domains of language and visuospatial abilities were considered to be impaired when patients abnormally performed both subtests of BOSU and the copy trial of the RCFT respectively.

According to this approach, all subjects were categorised into two subgroups: (i) ALS-ni: no cognitive impairment or (ii) ALS-ci: cognitive impairment in at least one cognitive domain.

Statistical analyses of neuropsychological data were performed with SPSS V.18. The healthy controls served as reference sample to categorise the patients. Depending on their parametric properties, group comparisons of selected dependent neuropsychological variables were conducted using a series of univariate analyses of covariance (ANCOVA with age and IQ as covariates), or nonparametric techniques (Welch-Tests or Kruskal-Wallis-Test), respectively. The significance level was set to p<0.05 and post hoc tests were adjusted for multiple comparisons using Bonferroni correction.

#### MRI data

The DTI datasets were processed with the FSL software package (www.fmrib.ox.ac.uk/fsl). Pre-processing included eddy currents and motion correction, average of two retry passes as well as brain-tissue extraction. After pre-processing, a diffusion tensor model was fitted at each voxel, generating maps of fractional anisotropy (FA), mean diffusivity (MD), axial diffusivity (AD: first eigenvalue), and radial diffusivity (RD: the average of the second and third eigenvalue).

Tract-based spatial statistics (TBSS [36]) was employed with a threshold of FA >0.2 for the mean FA skeleton. To compare diffusion measures between different patient groups and healthy controls, ANCOVAs were conducted considering age, gender and scanner site as covariates, using permutation tests with 5000 permutations and threshold-free cluster enhancement (TFCE) implemented in the FSL 4.1 submodule *Randomise*. We controlled for multiple comparisons by using the family-wise error (FWE) method (p<0.05).

In addition to the TBSS analysis, a region of interest (ROI) analysis was performed. To reduce the number of comparisons, we defined fifteen white matter tracts typically used in studies on ALS a priori, based on the ICBM-DTI-81-WM labels atlas (Johns Hopkins University, Baltimore, Maryland [37]): Body of corpus callosum (CC); corticospinal tract (CST-Left/Right); anterior corona radiata (ACR-L/R), sagittal stratum (SS-L/R, including both the inferior longitudinal fasciculus – ILF and inferior fronto-occipital fasciculus – IFOF); cingulum (CL-L/R); superior longitudinal fasciculus (SLF-L/R); superior fronto-occipital fasciculus (SFOF-L/R); and uncinate fasciculus (UF-L/R). Mean FA, MD, AD, and RD values within these tract labels in MNI space were extracted from the spatially normalized and skeletonized FA, MD, AD, and RD maps of each individual using custom Matlab code (MATLABR2007a). The DTI parameters of the ROIs were compared using ANCOVAs, with age, gender and scanner site as covariates (p<0.05). To adjust for multiple comparisons (number of ROIs), the Bonferroni-Holm-procedure was used.

Correlation analyses between the neuropsychological data and the DTI parameter values of the ROIs (excluding CST-L/R) were only performed in the patient sample. The relationship between neuropsychological test scores and the mean values of diffusivity parameters was evaluated using linear regression with stepwise selection (backward procedure with mean of diffusivity, age, gender and scanner site as predictors and neuropsychological values as dependent variables). For those variables without parametric properties, Spearman Rank correlations were performed. This analysis was conducted using SPSS V.18 with FDR-correction for multiple comparisons (Benjamin-Hochberg-Procedure) with a level of significance of p<0.05.

## Results

### Neuropsychological data

Based on the neuropsychological testing, 49 patients (68.1%) showed no cognitive impairment (ALS-ni) and 23 patients (31.9%) were cognitively impaired in at least one cognitive domain (ALS-ci). One healthy control subject was categorised as cognitively impaired and therefore excluded from further analyses. Comparisons including post hoc analysis of neuropsychological variables are presented in Table 3. ALS-ni-patients and ALS-ci-patients differed significantly in age, with ALS-ci being older than ALS-ni patients, but not in ALS-FRS-R and disease duration. Controls and ALS-ni patients differed significantly in one index of verbal fluency (RWT-G/R), in all self-rated FrSBe-Scales and in the family-rated FrSBe-Scale-Apathy. Compared to controls, the ALS-ci patients performed worse in almost all tasks, whereas the ALS-ci and ALS-ni-patients differed in only ca. 50% of the cognitive tests. Importantly, the variable Tower of London task revealed significant differences between controls and ALS-ni, with patients outperforming healthy controls.

**Table 3:** Comparison of dependent neuropsychological variables between cognitive subgroups and healthy controls.

	HC		ALS-ni		ALS-ci		Inter-group -differences p		
Dependent Variable	N	Mean	N	Mean	N	Mean	HC vs. ALS-ni	HC vs. ALS-ci	ALS-ci vs. ALS-ni
Age	64	**59.9**	49	**56.4**	23	**65.4**	0.163	0.066	**0.001** ^1^
ALSFRS			49	**36.3**	23	**37**			0.616^2^
Disease duration (m)			49	**34.5**	23	**22.1**			0.241^2^
**Executive functions**									
TMT^a^	64	**2.2**	43	**2.4**	21	**3.1**	1.000	**0.000**	0.002^1^
Stroop-I^a^	63	**1.11**	39	**1.14**	14	**1.26**	1.000	**0.020**	0.101^2,4^
Stroop-C^a^	62	**1.08**	38	**1.06**	13	**1.13**	1.000	1.000	0.561^2^
Stroop-E^a^	62	**0.81**	38	**1.29**	13	**3.46**	0.326	**0.035**	0.767^3,4^
RWT-K^a^	64	**3.68**	46	**3.64**	19	**5.93**	1.000	**0.000**	**0.000** ^2^
RWT-G/R^a^	64	**3.94**	45	**4.99**	19	**7.65**	**0.018**	**0.000**	**0.001** ^3^
RWT-A^a^	64	**1.82**	45	**1.76**	20	**3.2**	1.000	**0.000**	**0.000** ^2^
RWT-S/F^a^	64	**3.14**	45	**3.11**	19	**4.52**	1.000	**0.000**	**0.000** ^2^
DSB	64	**6.2**	49	**6**	22	**4.4**	0.835	**0.000**	**0.000** ^3^
ToL-E^a^	54	**0.8**	27	**1.2**	10	**0.4**	0.857	0.910	0.767^3^
ToL-M^a^	54	**10.1**	24	**8.5**	8	**6.5**	0.065	**0.002**	0.082^1^
**Memory**									
DSF	65	**7.6**	49	**8**	22	**6.4**	0.196	**0.002**	**0.000** ^1^
VMIR	64	**83.9**	49	**81.3**	21	**57.1**	1.000	**0.000**	**0.000** ^2^
VMDR	63	**82.8**	49	**75.4**	21	**52.4**	0.126	**0.000**	**0.000** ^2^
VMR	63	**85.9**	48	**80**	19	**64.5**	0.563	**0.002**	**0.049** ^2,4^
**Language**									
BOSU-P	64	**19.3**	44	**19.4**	15	**18.7**	0.933	**0.028**	**0.023** ^3^
BOSU-N	38	**19.9**	12	**19.8**	5	**19.6**	0.904	**0.037**	0.177^3^
**Visuo-spatial abilities**									
RCFT	49	**32.9**	29	**32.9**	17	**29.2**	1.000	**0.000**	**0.000^2^**
**Behaviour**									
FrSBe-F-A^a^	55	**23.2**	43	**28.9**	20	**28.5**	**0.000**	**0.007**	0.687^1^
FrSBe-F-D^a^	55	**22.9**	43	**24.4**	20	**22.2**	0.242	0.844	0.300^1^
FrSBe-S-D^a^	57	**22.5**	39	**25.8**	16	**25.8**	**0.005**	0.973	0.052^1^
FrSBe-F-E^a^	55	**30.2**	43	**31.9**	20	**31.1**	0.439	0.592	0.951^1^
FrSBe-S-E^a^	57	**27.8**	39	**32.2**	16	**30.33**	**0.007**	0.261	0.407^1^

a = higher scores indicate worse performance or high degree of abnormality respectively; ^1^ = analyzed by AN(C)OVA; ^2^ = analyzed by Welch-Test/ANOVA/Spearman rank correlations; ^3^ = analysed by Kruskal-Wallis-Test/U-Test/Spearman rank correlations; ^4^ **= **significant effect of the covariate age.

HC  =  Healthy Controls; ALS-ni =  ALS patients without cognitive impairment; ALS-ci  =  ALS-patients with cognitive impairment; TMT  =  Trail-Making Test; Stroop-I  =  Stroop-Inhibition; Stroop-C  =  Stroop-Congruence; Stroop-E  =  Total errors; RWT  =  Regensburg word fluency test; ToL-E  =  Tower of London-Number of rule breakings; ToL-M  =  Tower of London Ratio of number of moves and number of correct trials; VMIR  =  Verbal memory Immediate Recall; VMDR  =  Verbal Memory Delayed Recall; VMR  =  Verbal Memory Recognition; DSF/DSB  =  Digit Span forward and backward; BOSU-P  =  Bogenhausen Semantic Test –Pointing; BOSU-N  =  Bogenhausen Semantic Test –Naming; RCFT  =  Rey complex figure test; FrSBe  =  Frontal System Behaviour Scale; A  =  Apathy; D  =  Disinhibition; E  =  Executive Dysfunction; S  =  self rating; F  =  family rating.

### MRI data

#### Group comparisons of diffusivity values

**Figure 1:**
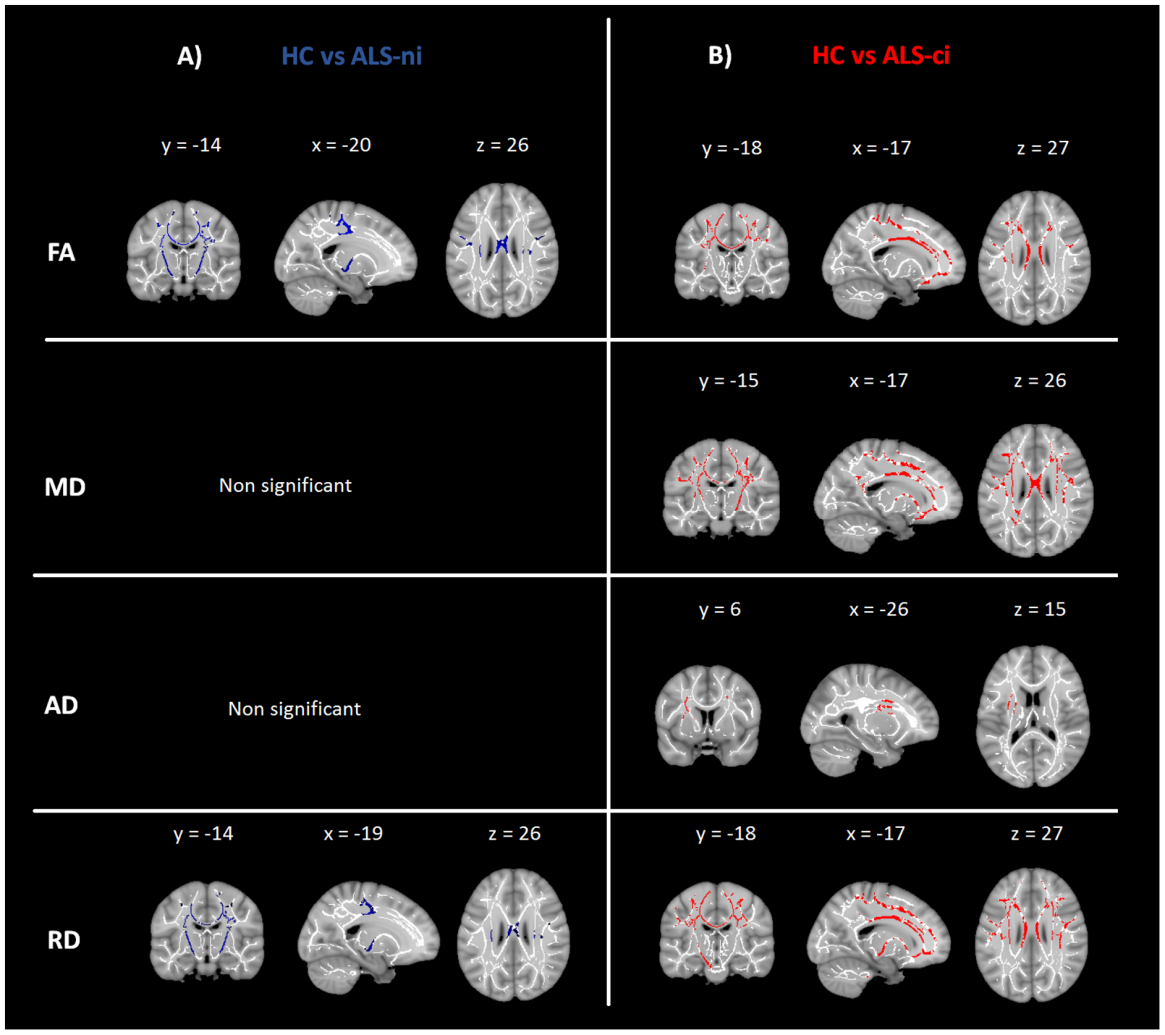
Group comparisons of diffusivity values between ALS patients and healthy controls. Effect of group differences along the TBSS fiber tract skeleton. Effects were thresholded at p<0.05, corrected for multiple comparisons. A) blue: comparison between cognitively intact ALS-patients to healthy controls; B) red  =  comparison between cognitively impaired ALS-patients to healthy controls; FA  =  fractional anisotropy; MD  =  mean diffusivity; AD  =  axial diffusivity; RD  =  radial diffusivity

In the TBSS analysis the FA and RD maps showed significant differences between ALS-ni-patients and healthy controls bilaterally in the corticospinal tract (CST), the posterior limb of the internal capsule, and in the body of the corpus callosum (CC) (Fig. 1A).

In addition to effects in the regions mentioned above, ALS-ci patients showed significantly decreased FA values as well as increased MD and RD values in the anterior thalamic radiation (ATR), the cingulum (CL), the inferior fronto-occipital fasciculus (IFOF), the superior and inferior longitudinal fasciculus (SLF/ILF), and in the uncinate fasciculus (UF) compared to controls. All changes occurred bilaterally but to a greater extent in the left hemisphere (Fig. 1B).

**Figure 2:**
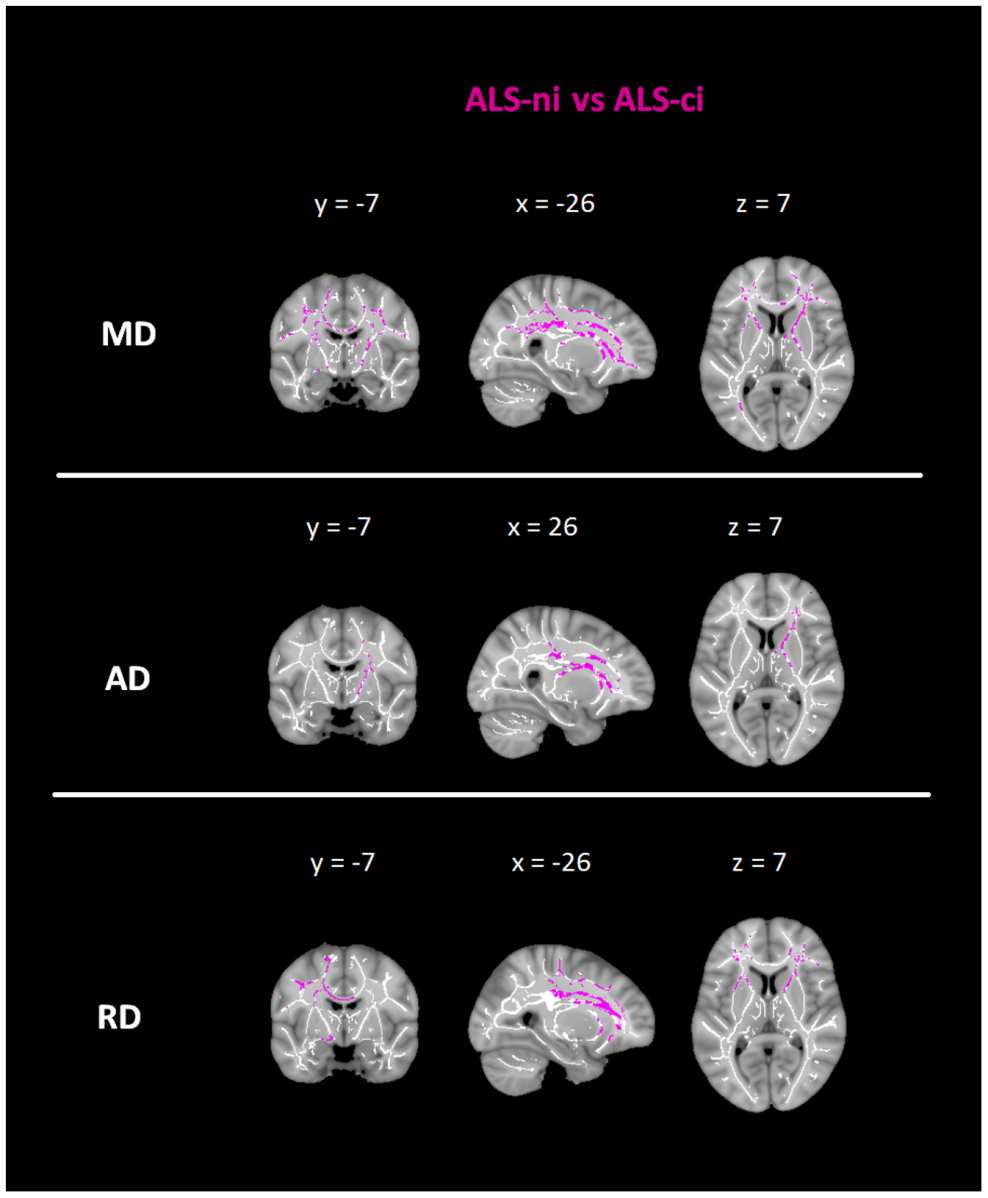
Group comparisons of diffusivity values between ALS-ni patients and ALS-ci patients. Effect of group differences along the TBSS fiber tract skeleton. Effects were thresholded at p<0.05, corrected for multiple comparisons. Violet: comparison between cognitively intact ALS-patients to cognitively impaired ALS-patients; MD  =  mean diffusivity; AD  =  axial diffusivity; RD  =  radial diffusivity

Significant differences between the ALS-ci-patients and the ALS-ni-patients were found regarding the MD and RD values bilaterally in the ATR, the CL, the IFOF, the SLF, and in the UF. Considering MD, significant differences were found in left temporal parts of these associative tracts. AD values differed significantly in the CR, the ATR, and the IFOF, with predominant effects for the right hemisphere (Fig. 2).

In the complementary ROI-analysis, only a few regions survived Bonferroni-Holm correction for multiple comparisons. Compared to controls, the ALS-ni patients showed significantly decreased FA within the right CST (p = 0.001). Compared to controls, the ALS-ci-patients showed significantly decreased FA and increased RD within the left CST (p = 0.001). No significant differences were found between ALS-ni patients and ALS-ci patients. A full list of all comparisons, including means and standard deviations of all regions, is presented in S1 Table.

#### Correlations of diffusivity values with neuropsychological variables

Table 4 shows the significant correlations of diffusivity values with neuropsychological variables within the ALS patient sample. We present the correlation parameters of the diffusivity values with the highest correlation score including statistical values. Significant correlations of other diffusivity parameters are listened only (table 4 last column). A few cognitive scores derived from executive and memory tests correlated consistently with selected white matter areas, each in the neurobiologically expected direction. Alterations of white matter tracts were found in both commissural tracts (CC) and in associative tracts (ACR, SFOF, and UF). The majority of cognitive scores correlated with the radial diffusivity. Regarding behaviour, the FrSBe subscale *Executive dysfunctions* correlated significantly with left and right SLF and right SFOF.

**Table 4:** Significant correlations of diffusivity values with neuropsychological variables in patients with ALS.

NPS Variable	ROI		Diffusivity value with the highest correlation score^1^						Other diffusivity values with significant correlations
				ß	Rsqare	r_s_	p	p_FDR_	
**RWT-A**	CC-B		**AD**			0.411	0.001	**0.02**	
**Stroop-I**	CC-B		**AD**	0.428	0.183		0.001	**0.02**	
	ACR	R	**FA**	−0.433	0.188		0.001	**0.02**	*MD,RD*
		L	**RD**	0.382	0.146		0.005	**0.02**	*FA,MD*
	CL	R	**MD**	0.409	0.167		0.002	**0.02**	*RD*
		L	**RD**	0.416	0.173		0.002	**0.02**	*FA*
	SFOF	R	**MD**	0.353	0.125		0.009	**0.03**	
		L	**RD**	0.393	0.155		0.004	**0.02**	*FA, MD*
	UF	R	**MD**	0.493	0.243		0.000	**0.01**	*AD,RD*
**DSB**	CC-B		**AD**	−0.331	0.110		0.005	**0.04**	
	ACR	R	**MD**	−0.309	0.095		0.009	**0.04**	
		L	**FA**	0.358	0.128		0.002	**0.03**	*RD MD*
	SFOF	L	**AD**	−0.333	0.111		0.005	**0.04**	*MD*
**VMDR**	ACR	L	**FA**	0.362	0.132		0.002	**0.04**	
**VMR**	CC-B		**AD**	−0.370	0.137		0.002	**0.03**	*MD*
	ACR	R	**MD**	−0.323	0.104		0.008	**0.04**	*RD*
		L	**MD**	−0.363	0.132		0.003	**0.04**	*RD*
	UF	R	**RD**	−0.315	0.099		0.009	**0.04**	
		L	**RD**	−0.309	0.095		0.011	**0.04**	
**RCFT**	CC-B		**MD**	−0.376	0.134		0.012	**0.04**	
	ACR	R	**RD**	−0.472	0.222		0.001	**0.01**	*FA, MD*
		L	**RD**	−0.528	0.278		0.000	**0.01**	*FA, MD*
	SFOF	L	**RD**	−0.370	0.137		0.011	**0.03**	*MD*
	UF	R	**RD**	−0.395	0.156		0.007	**0.02**	*MD*
**FrSBe-F-E**	SLF	L	**RD**	0.382	0.146		0.002	**0.03**	
**FrSBe-S-E**	SLF	R	**FA**	−0.401	0.161		0.004	**0.03**	
		L	**FA**	−0.384	0.384		0.004	**0.03**	
	SFOF	R	**AD**	0.449	0.201		0.001	**0.01**	*MD*

1 including results of regression analysis and Spearman-rank-correlation respectively (p^FDR^<0.05)

ROI  =  Region of interest; Stroop-I  =  Stroop-Inhibition; VMDR  =  Verbal Memory Delayed recall; VMR  =  Verbal Memory Recognition; DSB  =  Digit span backward; RCFT  =  Rey complex figure test; FrSBe  =  Frontal System Behaviour Scale; E  =  Executive Dysfunction; S  =  self rating; F  =  family rating; FA  =  fractional anisotropy; MD  =  mean diffusivity; AD  =  axial diffusivity RD  =  radial diffusivity; L  =  Left; R  =  Right; CC-B  =  Body of corpus callosum; CST  =  corticospinal tract; ACR  =  anterior corona radiate; SS  =  sagittal stratum including both the inferior longitudinal fasciculus (ILF) and inferior fronto-occipital fasciculus(IFOF)); CL  =  cingulum; SLF  =  superior longitudinal fasciculus; SFOF  =  superior fronto-occipital fasciculus; UF  =  uncinate fasciculus.

## Discussion

In this study, we investigated a large cohort of ALS patients, including subjects with cognitive impairments, to determine the relationship of cognitive and behavioural alterations to white matter microstructural integrity as quantified by DTI. Our study presents data from a relatively large sample of patients with ALS and healthy controls; it extends findings from previous studies that were based on smaller sample sizes and less comprehensive neuropsychological characterisation of subjects.

Based on neuropsychological testing, the majority of ALS patients (around 70%) showed no cognitive impairment (ALS-ni), while around 30% were cognitively impaired in at least one cognitive domain (ALS-ci). The proportion of cognitive impairment is in line with reports from the literature [2]. ALS-ni patients showed only a reduced verbal fluency (RWT-G/R) in comparison to controls. This agrees with the criteria of Strong et al. 2009 [33] and Phukan et al. 2012 [35], which require impairments in at least two cognitive tests before a subject is categorised as cognitively impaired. ALS-ni patients reported a significantly higher magnitude of abnormal behaviour with increased apathy, disinhibition, and dysexecutive behaviour than did the controls. Increased levels of apathy in ALS-ni patients were also confirmed by the family ratings. Patients classified as cognitively impaired (ALS-ci) performed worse in almost all individual tests compared with controls. ALS-ci and ALS-ni patients, however, differed from each other in only about half of all cognitive tests, whereby test differences were observed in multiple domains, such as executive functions, memory and language. Interestingly, in the Tower of London task, an indicator of problem solving skills, patients showed significantly better results in the number of moves (ToL-M). This variable is an indicator for ability to form effective problem solving strategies [38]. A possible explanation for this is that at the behavioural level patients with ALS preferred a slower, more careful approach to these tasks due to their physical impairments.

In line with previous findings, patients without cognitive impairment (ALS-ni) demonstrated the characteristic pattern of ALS-related predominant white matter changes in the corticospinal tract and the body of the corpus callosum [39]. In addition, the cognitively impaired patients (ALS-ci) showed significant white matter changes within extra-motor regions, particularly the frontal areas or frontal-temporal association fibres (anterior thalamic radiation, cingulum, inferior fronto-occipital fasciculus, superior longitudinal fasciculus, and uncinate fasciculus). Importantly, the direct comparison of the ALS-ni and ALS-ci groups revealed significant differences in white matter fiber tracts in these same regions. This effect remained despite the relatively large age difference between the subgroups (ALS-ni = 56.4 years vs. ALS-ci = 65.4 years). These results suggest that an involvement of extra-motor areas underlies the impairment in cognition in a relevant fraction of nondemented ALS patients. Furthermore, the significant differences in the few extra-motor areas observed in the comparison between ALS-ni patients and healthy controls may indicate white matter changes in ALS-ni patients preceding manifest cognitive and behavioural changes. Thus, some of the ALS-ni patients could already show subtle brain changes without cognitive symptoms.

Nevertheless, the ROI analysis yielded no effects beyond the motor system and the corpus callosum. This may be caused by the parcellation approach we had adopted, which only provides DTI parameters for the selected fibre tracts. Averaging across an entire tract renders this approach less sensitive to focal changes within a tract. Support for asymptomatic brain changes comes from a functional MRI study by Goldstein [19], showing altered brain activation during an interference task in the absence of changes in test performance in patients with ALS versus healthy controls. Another study involving cognitively unimpaired patients found grey matter atrophy in frontal regions [40]. Longitudinal studies are necessary to help resolve the question of whether structural brain changes precede cognitive symptoms in cognitively unimpaired ALS patients.

In addition, our findings may shed further light on the “disease continuum vs. distinct disease trajectories” controversy. Thus, neuropathological and clinical overlaps may indicate a continuum between ALS and fronto-temporal dementia (FTD). The majority of cognitively impaired patients show executive dysfunctioning or language deficits, similar to the pattern occurring within the spectrum of FTD, but to a lower extent. However, our findings of a fronto-temporal pattern of white matter disintegrity as well as differences in a few extra-motor areas observed in the comparison between ALS-ni patients and healthy controls may confirm the hypothesis of a continuum. Our second analysis focused on the correlation of cognitive variables and white matter abnormalities. Taking patient's age into account, we found significant correlations between executive and memory tasks and white matter abnormalities, with decreased FA values and increased MD, AD, or RD values. Significant correlations were mainly found for executive tasks. Thus, impaired response inhibition in the Stroop-task was associated with disintegrity of predominantly frontal (ACR), fronto-temporal (CL, UF), and fronto-occipital (SFOF) fibre bundles. Moreover, the digit-span backward task, which measures the ability to mentally manipulate information within the phonological loop processes of working memory [41], was associated with these aforementioned structures.

Surprisingly, after FDR correction, verbal fluency did not correlate noticeably with any tract, even though this was demonstrated in a previous study [42]. This may be caused by the parcellation approach, as explained above. This approach is less sensitive to focally located functions such as verbal fluency, which is functionally specific to the inferior frontal gyrus [43]. But our statistical approach is more comparable with the study of Sarro et al. [12], who also failed to find significant correlations between DTI parameters and verbal fluency scores after correction for multiple comparisons.

In terms of the other cognitive domains, the error-corrected recognition (VMR) as part of a verbal memory task also contains executive demands [44]. It is, therefore, reasonable to assume that fronto-temporal association tracts are involved (e.g., anterior corona radiata). In addition, the VMR correlated with the uncinate fasciculus, which is involved in decision making, learning, and memory, an ability required in the recognition condition of this task [20], [45]. Within the domain of visuo-spatial abilities, we found an effect for the *Rey complex figure test* (RCFT), which can be explained by the test properties itself. This test encompasses visuo-spatial abilities as well as executive functioning [46], potentially accounting for the involvement of long association tracts (e.g. SFOF).

The results of the regression analyses are relatively comparable with the existing literature. Similar to our findings, Abrahams et al. 2005 [11] also found correlations between the corona radiata and different cognitive tasks (e.g. short term memory span, paired associate learning). Petitt et al. [17] used a dual task paradigm to assess working memory, comparable to our digit span backward task, and revealed significant correlations with the corona radiata. Our study also replicates findings by Sarro et al. [12], who employed a comparable test procedure. The consistent pattern of significant correlations between executive functions and white matter fiber tract integrity in our study confirms and extends these previous results in a larger patient sample using a rather conservative correction for multiple comparisons.

This investigation addressed an important gap in the literature, as it integrated behavioural scores into the analysis. Contrary to our hypothesis based on previous studies [21], [22], FrsBe-Scale apathy did not significantly correlate with any of the investigated white matter tracts, which may be related to differences between methods in those studies and ours. For example, there were differences in terms of the data acquisition (3 Tesla versus 4 Tesla [22]) and the use of conservative vs. liberal significance-levels (p<0.05 corrected for multiple comparisons in our study versus p<0.001 uncorrected for multiple comparisons [21]). Further studies are required to resolve these inconsistencies. Interestingly, the FrSBe-Scale *Executive dysfunction*, which represents daily associated executive dysfunctions, revealed significant correlations with the large association fibres. This finding is consistent with prior work on white matter changes associated with single executive cognitive tasks [11], [12].

In addition, our study demonstrates that the integration of different DTI-metrics is useful when investigating ALS and the neurocorrelates of changes in behavioural measurements due to the disorder. Our results indicate radial diffusivity as the most sensitive marker in this context, confirming previous findings [47]. The discussion about the pathological substrate underlying the DTI metrics remains open, in particular as the distinction of parts of axonal architecture is difficult to achieve [48]. It is common knowledge that FA is believed to reflect the eccentricity of fibre bundles respectively the degree of myelination and axonal density [49]. FA appears sensitive to fibres which are aligned over a large enough scale like corpus callosum, cortico-spinal tarct or optic tract. MD appears to be a measure of the average molecular motion independent of any tissue directionality. AD as the primary eigenvector describes water diffusivity in the direction of the fiber tract and assesses axonal functions or axonal degeneration respectively. RD reflects diffusivity perpendicular to axonal fibres and is more sensitive to myelin abnormalities. The metric appears to reflect a loss of integrity of the axonal wall, highly sensitive to neurodegeneration [48].

Considering our data, at present, we are not able to draw clear conclusions about the pathological substrate underlying cognitive impairment in ALS for methodological reasons: FA describes a quotient and MD describes a mean, so that mild alterations in single metrics cancel each other out. Only, if analysing single metrics, we did find changes. However, using various DTI metrics appears to be worthwhile.

One limitation of our study arises from the small subgroups, despite the rather large total sample. In addition, the level of cognitive impairment varied greatly within the group of cognitively impaired patients. Our sample contained patients ranging from those with barely perceptible impairments just below the average to impairments which patients notice in their daily activities or caregivers report. Further, it is worth noting that most of the neuropsychological variables and the results of the DTI metrics were confounded by the covariate age and that the ALS-ni patient group was significantly younger. However, as we controlled for these nuisance effects in the statistical model, we are allowed to make specific conclusions. Nevertheless, the effects, were maintained after controlling for age.

In summary, in a large cohort of non-demented ALS patients we found white matter changes in relation to executive dysfunctioning and memory disturbances. The white matter characteristics of cognitively impaired patients differed significantly from healthy controls in both motor and extra-motor areas with a predominant involvement of long association fibre bundles, including prefrontal and temporal regions. Notably, the direct comparison of the ALS-ni and ALS-ci groups revealed significant differences in white matter fiber tracts in these regions.

In particular, correlations between executive tasks and functionally corresponding white matter structures suggest that cognitive impairment is related to structural white matter changes in ALS without dementia.

## Supporting Information

S1 Table **Comparison of ROIs between cognitive subgroups and healthy controls with age, gender and scanner site as covariates.** ROI  =  Region of interest; HC  =  Healthy Controls; ALS-ni =  ALS patients without cognitive impairment; ALS-ci  =  ALS-patients with cognitive impairment; FA  =  fractional anisotropy; MD  =  mean diffusivity; AD  =  axial diffusivity RD  =  radial diffusivity; L  =  Left; R  =  Right; CC-B  =  Body of corpus callosum; CST  =  corticospinal tract; ACR  =  anterior corona radiate; SS  =  sagittal stratum including both the inferior longitudinal fasciculus and inferior fronto-occipital fasciculus); CL  =  cingulum; superior longitudinal fasciculus (SLF-L/R); SFOF  =  superior fronto-occipital fasciculus; UF  =  uncinate fasciculus *surviving Bonferroni-Holm correction for multiple comparisons (p<0.05).
(DOCX)
