# Supplementary material for: Microstructural White Matter Changes Underlying Cognitive and Behavioural Impairment in ALS – An In Vivo Study Using DTI
Source: PLoS One. 2014 Dec 11;9(12):e114543. doi: 10.1371/journal.pone.0114543 (PMC4263750; doi:10.1371/journal.pone.0114543)
Supplement: S1 Table — Comparison of ROIs between cognitive subgroups and healthy controls with age, gender and scanner site as covariates. ROI = Region of interest; HC = Healthy Controls; ALS-ni = ALS patients without cognitive impairment; ALS-ci = ALS-patients with cognitive impairment; FA = fractional anisotropy; MD = mean diffusivity; AD = axial diffusivity RD = radial diffusivity; L = Left; R = Right; CC-B = Body of corpus callosum; CST = corticospinal tract; ACR = anterior corona radiate; SS = sagittal stratum including both the inferior longitudinal fasciculus and inferior fronto-occipital fasciculus); CL = cingulum; superior longitudinal fasciculus (SLF-L/R); SFOF = superior fronto-occipital fasciculus; UF = uncinate fasciculus *surviving Bonferroni-Holm correction for multiple comparisons (p<0.05). (DOCX) [file pone.0114543.s001.docx]

*Table S1*

*Comparison of ROIs between cognitive subgroups and healthy controls with age, gender and scanner site as covariates*

|  | | **HC** |  | **ALS-ni** |  | **ALS-ci** |  | **Inter-group-differences** |  |  |
| --- | --- | --- | --- | --- | --- | --- | --- | --- | --- | --- |
| **ROI** | | Mean | SD | Mean | SD | Mean | SD | HC vs. ALS-ni | HC vs. ALS-ci | ALS-ni vs. ALS-ci |
|  | | | | | | | | p | p | p |
| **FA** | | | | | | | | | | |
| CST | R | 0.61 | 0.04 | 0.59 | 0.04 | 0.58 | 0.04 | **0.001*** | 0.018 | 0.832 |
|  | L | 0.63 | 0.04 | 0.60 | 0.04 | 0.61 | 0.04 | 0.058 | **0.000*** | 0.015 |
| CC-B |  | 0.70 | 0.04 | 0.70 | 0.04 | 0.68 | 0.05 | 0.108 | 0.007 | 0.163 |
| ACR | R | 0.48 | 0.03 | 0.48 | 0.04 | 0.46 | 0.04 | 0.607 | 0.142 | 0.314 |
|  | L | 0.46 | 0.03 | 0.46 | 0.03 | 0.44 | 0.04 | 0.959 | 0.021 | 0.033 |
| SS | R | 0.56 | 0.03 | 0.57 | 0.03 | 0.55 | 0.03 | 0.869 | 0.717 | 0.826 |
|  | L | 0.55 | 0.03 | 0.56 | 0.03 | 0.55 | 0.04 | 0.930 | 0.662 | 0.729 |
| CL | R | 0.61 | 0.03 | 0.61 | 0.05 | 0.60 | 0.04 | 0.607 | 0.469 | 0.294 |
|  | L | 0.64 | 0.03 | 0.64 | 0.04 | 0.63 | 0.05 | 0.718 | 0.725 | 0.554 |
| SLF | R | 0.52 | 0.02 | 0.51 | 0.03 | 0.51 | 0.03 | 0.470 | 0.610 | 0.962 |
|  | L | 0.52 | 0.02 | 0.51 | 0.03 | 0.51 | 0.03 | 0.408 | 0.546 | 0.973 |
| SFOF | R | 0.53 | 0.04 | 0.53 | 0.05 | 0.52 | 0.04 | 0.494 | 0.822 | 0.775 |
|  | L | 0.52 | 0.06 | 0.50 | 0.05 | 0.50 | 0.07 | 0.336 | 0.471 | 0.981 |
| UF | R | 0.56 | 0.05 | 0.57 | 0.04 | 0.55 | 0.05 | 0.496 | 0458 | 0.235 |
|  | L | 0.53 | 0.04 | 0.52 | 0.05 | 0.52 | 0.02 | 0.641 | 0.417 | 0.672 |
| **MD (x 10^-3^ mm^2^ s^-1^)** | | | | | | | | | | |
| CST | R | 0.62 | 0.03 | 0.62 | 0.04 | 0.61 | 0.04 | 0.857 | 0.736 | 0.799 |
|  | L | 0.60 | 0.04 | 0.61 | 0.04 | 0.61 | 0.03 | 0.614 | 0.205 | 0.410 |
| CC-B |  | 0.76 | 0.04 | 0.77 | 0.04 | 0.80 | 0.05 | 0.673 | **0.049** | 0.121 |
| ACR | R | 0.73 | 0.03 | 0.72 | 0.04 | 0.75 | 0.04 | 0.684 | **0.032** | **0.021** |
|  | L | 0.73 | 0.03 | 0.73 | 0.04 | 0.76 | 0.05 | 0.641 | **0.039** | **0.024** |
| SS | R | 0.78 | 0.04 | 0.78 | 0.04 | 0.79 | 0.04 | 0.988 | 0.593 | 0.609 |
|  | L | 0.78 | 0.03 | 0.78 | 0.04 | 0.79 | 0.03 | 0.976 | 0.831 | 0.824 |
| CL | R | 0.67 | 0.03 | 0.67 | 0.03 | 0.68 | 0.03 | 0.106 | 0.816 | 0.164 |
|  | L | 0.68 | 0.03 | 0.68 | 0.03 | 0.69 | 0.03 | 0.040 | 0.959 | 0.147 |
| SLF | R | 0.71 | 0.03 | 0.71 | 0.03 | 0.73 | 0.03 | 0.915 | 0.108 | 0.113 |
|  | L | 0.71 | 0.03 | 0.71 | 0.03 | 0.72 | 0.02 | 0.593 | 0.184 | 0.389 |
| SFOF | R | 0.63 | 0.04 | 0.64 | 0.05 | 0.66 | 0.05 | 0.263 | 0.128 | 0.535 |
|  | L | 0.63 | 0.05 | 0.65 | 0.06 | 0.67 | 0.07 | 0.114 | 0.120 | 0.756 |
| UF | R | 0.68 | 0.04 | 0.67 | 0.04 | 0.70 | 0.05 | 0.582 | 0.091 | **0.048** |
|  | L | 0.71 | 0.04 | 0.71 | 0.04 | 0.72 | 0.04 | 0.864 | 0.462 | 0.415 |
| **AD (x 10^-3^ mm^2^ s^-1^ )** | | | | | | | | | | |
| CST | R | 1.08 | 0.06 | 1.06 | 0.06 | 1.05 | 0.06 | **0.005** | **0.040** | 0.880 |
|  | L | 1.08 | 0.06 | 1.07 | 0.07 | 1.05 | 0.04 | 0.489 | 0.116 | 0.328 |
| CC-B |  | 1.55 | 0.05 | 1.55 | 0.05 | 1.56 | 0.05 | 0.085 | 0.328 | 0.734 |
| ACR | R | 1.15 | 0.04 | 1.14 | 0.04 | 1.16 | 0.04 | 0.174 | 0.262 | **0.042** |
|  | L | 1.13 | 0.04 | 1.12 | 0.04 | 1.14 | 0.05 | 0.397 | 0.623 | 0.283 |
| SS | R | 1.32 | 0.05 | 1.32 | 0.05 | 1.33 | 0.05 | 0.753 | 0.958 | 0.781 |
|  | L | 1.32 | 0.04 | 1.32 | 0.05 | 1.33 | 0.04 | 0.910 | 0.961 | 0.971 |
| CL | R | 1.21 | 0.05 | 1.20 | 0.05 | 1.21 | 0.05 | 0.265 | 0.582 | 0.769 |
|  | L | 1.27 | 0.05 | 1.25 | 0.06 | 1.27 | 0.07 | 0.138 | 0.667 | 0.499 |
| SLF | R | 1.15 | 0.04 | 1.15 | 0.05 | 1.18 | 0.04 | 0.519 | 0.142 | 0.066 |
|  | L | 1.14 | 0.04 | 1.14 | 0.03 | 1.16 | 0.04 | 0.907 | 0.190 | 0.189 |
| SFOF | R | 1.05 | 0.06 | 1.06 | 0.06 | 1.09 | 0.06 | 0.436 | 0.090 | 0.303 |
|  | L | 1.03 | 0.07 | 1.04 | 0.08 | 1.07 | 0.08 | 0.246 | 0.298 | 0.893 |
| UF | R | 1.16 | 0.05 | 1.16 | 0.06 | 1.19 | 0.06 | 0.912 | 0.211 | 0.274 |
|  | L | 1.18 | 0.06 | 1.17 | 0.06 | 1.18 | 0.04 | 0.463 | 0.969 | 0.569 |
| **RD (x 10^-3^ mm^2^ s^-1^)** | | | | | | | | | | |
| CST | R | 0.38 | 0.03 | 0.40 | 0.04 | 0.40 | 0.04 | **0.042** | 0.050 | 0.716 |
|  | L | 0.36 | 0.04 | 0.38 | 0.04 | 0.39 | 0.04 | 0.154 | **0.002*** | 0.052 |
| CC-B |  | 0.38 | 0.05 | 0.39 | 0.05 | 0.42 | 0.06 | 0.249 | **0.011** | 0.119 |
| ACR | R | 0.52 | 0.04 | 0.52 | 0.04 | 0.55 | 0.05 | 0.870 | **0.031** | 0.057 |
|  | L | 0.53 | 0.04 | 0.53 | 0.04 | 0.57 | 0.05 | 0.865 | **0.012** | **0.014** |
| SS | R | 0.51 | 0.04 | 0.50 | 0.04 | 0.53 | 0.04 | 0.845 | 0.454 | 0.575 |
|  | L | 0.51 | 0.04 | 0.51 | 0.04 | 0.52 | 0.04 | 0.978 | 0.761 | 0.791 |
| CL | R | 0.40 | 0.03 | 0.40 | 0.04 | 0.41 | 0.04 | 0.306 | 0.498 | 0.169 |
|  | L | 0.39 | 0.03 | 0.40 | 0.04 | 0.38 | 0.04 | 0.291 | 0.771 | 0.299 |
| SLF | R | 0.49 | 0.03 | 0.49 | 0.03 | 0.50 | 0.03 | 0.787 | 0.247 | 0.373 |
|  | L | 0.49 | 0.03 | 0.49 | 0.04 | 0.50 | 0.03 | 0.470 | 0.358 | 0.735 |
| SFOF | R | 0.42 | 0.04 | 0.43 | 0.05 | 0.44 | 0.05 | 0.285 | 0.290 | 0.830 |
|  | L | 0.43 | 0.05 | 0.44 | 0.05 | 0.46 | 0.08 | 0.138 | 0.129 | 0.726 |
| UF | R | 0.44 | 0.05 | 0.43 | 0.04 | 0.46 | 0.06 | 0.448 | 0.182 | 0.073 |
|  | L | 0.48 | 0.05 | 0.49 | 0.05 | 0.50 | 0.05 | 0.841 | 0.402 | 0.522 |

ROI = Region of interest; HC = Healthy Controls; ALS-ni= ALS patients without cognitive impairment; ALS-ci = ALS-patients with cognitive impairment; FA = fractional anisotropy; MD = mean diffusivity; AD = axial diffusivity RD = radial diffusivity; L = Left; R = Right; CC-B = Body of corpus callosum; CST = corticospinal tract; ACR = anterior corona radiate; SS = sagittal stratum including both the inferior longitudinal fasciculus and inferior fronto-occipital fasciculus); CL = cingulum; superior longitudinal fasciculus (SLF-L/R); SFOF = superior fronto-occipital fasciculus; UF = uncinate fasciculus

*surviving Bonferroni-Holm correction for multiple comparisons (p<0.05)
